# A Successful Treatment Regimen for the Prevention of Sinusitis after Maxillary Sinus Floor Elevation Surgery in a High-Risk Case

**DOI:** 10.1155/2020/6869805

**Published:** 2020-08-07

**Authors:** Mikiko Suzuki-Yamazaki, Keiso Takahashi, Satoshi Takada, Yasumasa Kato, Yuh Baba

**Affiliations:** ^1^Department of Conservative Dentistry, Ohu University School of Dentistry, 31-1 Misumido, Tomita-machi, Koriyama City, Fukushima 963-8611, Japan; ^2^Department of Oral and Maxillofacial Surgery, Ohu University School of Dentistry, 31-1 Mitsumido, Tomita-machi, Koriyama City, Fukushima 963-8611, Japan; ^3^Department of Oral Function and Molecular Biology, Ohu University School of Dentistry, 31-1 Misumido, Tomita-machi, Koriyama City, Fukushima 963-8611, Japan; ^4^Department of General Clinical Medicine, Ohu University School of Dentistry, 31-1 Misumido, Tomita-machi, Koriyama City, Fukushima 963-8611, Japan

## Abstract

Maxillary sinus floor elevation (sinus lift) is a widely recognized dental-surgical approach for dental implant placement. However, for an otorhinolaryngological high-risk patient with severe anatomic-structural impairments of the maxillary sinus drainage pathway, surgical intervention is recommended before sinus lift to avoid postsinus lift maxillary sinusitis. Here, we show a case that postsinus lift maxillary sinusitis in such a high-risk patient was noninvasively prevented by the collaboration of otorhinolaryngologist and dentist. A 48-year-old Japanese male intended to undergo a sinus lift for dental implant placement by periodontist. Otorhinolaryngologist found septal deviation, concha bullosa, the presence of Haller cell, and nasal mucosal swelling by the nasal allergy, while no sinusitis and diagnosed him as a “high-risk case” for postsinus lift maxillary sinusitis. The patient was administered preoperative topical steroid and leukotriene receptor antagonist in addition to perioperative antibiotic prophylaxis so that his complication was noninvasively prevented. Thus, this case suggested that consultation from dentist to otorhinolaryngologist provides benefit to the patients who have been diagnosed as “high-risk case” for postsinus lift maxillary sinusitis.

## 1. Introduction

Maxillary sinus floor elevation (sinus lift) introduced by Boyne and James [1] is a widely recognized dental surgery to create a mucoperiosteal pocket over the maxillary floor and beneath the Schneider's membrane in which to place the bone graft material or dental implant to rehabilitate the upper dental arch, in the atrophic maxilla. Although sinus lift is an effective approach, maxillary sinusitis sometimes occurs as a complication after surgery [2, 3]. Anatomic-structural impairments of the maxillary sinus drainage pathway such as septal deviation, concha bullosa, the presence of Haller cell, and nasal mucosal swelling owing to nasal allergy increase in the incidence of maxillary sinusitis after sinus lift [4]. Thus, these patients are diagnosed as a “reversible contraindication case” or “high-risk case” in the field of otorhinolaryngology for the sinus lift [5, 6]. For the patient with severe anatomic-structural impairments of the maxillary sinus drainage pathway, surgical intervention is recommended before sinus lift to avoid postsinus lift maxillary sinusitis.

If patients do not agree with the surgical treatment, “noninvasive treatment” with reducing maxillary sinusitis incidence safely should provide great benefit. Here, we report a successful treatment modality for the noninvasive prevention of sinusitis after the sinus lift in an otorhinolaryngological high-risk case.

## 2. Case Report

**Figure 1:**
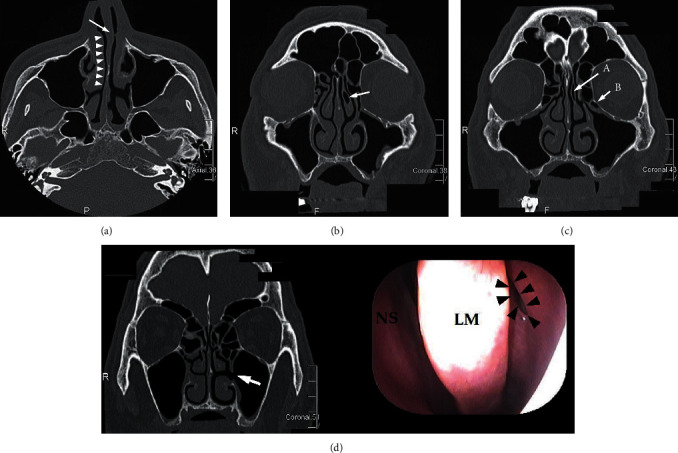
Preoperative CT: (a) left nasal septal deviation (arrow) and swelling of right inferior turbinate (arrowheads); (b) left concha bullosa of middle turbinate (arrow); (c) the narrow of left maxillary sinus ostium owing to the left concha bullosa of middle turbinate (arrow A) and left Haller cell (arrow B). (d) Left accessory ostium of maxillary sinus (arrow in CT (left) and arrowheads in nasal endoscopic examination (right). NS, nasal septum; LM, left middle turbinate.

A 48-year-old Japanese male came to otorhinolaryngology in our hospital. Although periodontist (S-Y, M) planned bilateral sinus lift and implant placement to him, he has complained of alternating nasal congestion for one year. Thus, he consulted an otorhinolaryngologist (BY). There was no history of any operation in the head and neck, and he had a nasal allergy. Preoperative computer tomography (CT) and nasal endoscopic examination indicated septal deviation, concha bullosa, the presence of Haller cell, nasal mucosal swelling owing to nasal allergy, and maxillary sinus accessory ostium, while no sinusitis was detected (Figure 1). Therefore, the patient was judged as a reversible otorhinolaryngological contraindication to a sinus lift (a high-risk case). After we explained whether he received presinus lift medical conservative therapy or presinus lift surgical treatment such as functional endoscopic sinus surgery (FESS) in order to prevent maxillary sinusitis after sinus lift, he chose medical conservative therapy. Thus, we preoperatively prescribed both topical nasal steroid (fluticasone propionate aqueous nasal spray) and leukotriene receptor antagonist (pranlukast hydrate) for him. Two months later, nasal congestion almost disappeared, and he underwent bilateral maxillary sinus floor elevation with bone graft material (Cytrans Granules®, GC company, Tokyo, Japan) by using sinus lift technique.

**Figure 2:**
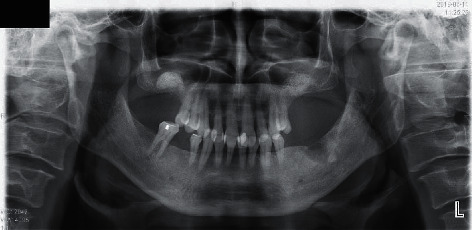
No maxillary sinusitis and the graft material of bilateral maxillary sinus floor was detected in postoperative panoramic radiography.

After surgery, no clinical symptoms had occurred. No maxillary sinusitis was found in follow-up panoramic radiography at 2 months after bilateral sinus lifts (Figure 2), and the postoperative course was uneventful before his 7 months follow-up evaluation. He will undergo bilateral implant placement in other local dental clinics.

## 3. Discussion

Pignatro et al. proposed that otorhinolaryngological contraindications to a sinus lift can be divided into those presumably irreversible and those that are potentially reversible [5, 6]. The contraindications were categorized into irreversible and reversible ones. Irreversible contraindications involve Wegener's granulomatosis, sarcoidosis, inverted papilloma, and malignant nasosinus neoplasms involving the maxillary sinus and/or the adjacent anatomic structures, and reversible contraindications involve anatomic-structural impairments of the maxillary sinus drainage pathways and phlogistic-infective processes. Thus, otorhinolaryngologist (BY) diagnosed that this case was a reversible contraindication because he had septal deviation, concha bullosa, the presence of Haller cells, nasal mucosal swelling owing to nasal allergy, and maxillary sinus accessory ostium. Thus, we presented to him whether he received presinus lift medical conservative therapy or presinus lift surgical treatment. Appropriate surgical treatment of this case was considered to be functional endoscopic sinus surgery (FESS) + septoplasty + turbinectomy or minimally invasive endoscopic middle meatal antrostomy proposed by Kunihiro et al. [7]. Because the patient did not like more than one surgery, he selected medical conservative therapy.

Medical conservative therapy for the prevention of maxillary sinusitis after sinus lift includes perioperative antibiotic prophylaxis and preoperative administration of topical steroid [6]. Lee et al. mentioned that the use of nasal decongestants should be considered to reduce the risk of postoperative sinusitis [4]. However, we did not use vasoconstrictor because the use of it might further compromise the already low oxygen tension in the sinus [8]. Instead of vasoconstrictor, we prescribed a leukotriene receptor antagonist for him because he had nasal mucosal swelling owing to nasal allergy. Consequently, the complications such as maxillary sinusitis did not occur after sinus lift.

In summary, we reported a successful treatment regimen to prevent sinusitis after maxillary sinus floor elevation in a high-risk case. Administration of preoperative topical steroid and leukotriene receptor antagonist in addition to perioperative antibiotic prophylaxis (Sawacillin®) may be effective for the prevention of sinusitis after sinus lift as medical conservative therapy to high-risk cases who had both anatomic-structural impairments of the maxillary sinus drainage pathways and nasal allergy although there is certain limitation. Therefore, further clinical trial is required to insist on our “noninvasive” treatment regimen, which is a great advantage for the patient, to regulate the inflammatory reaction of nasal mucosa in such high-risk cases. Furthermore, this case suggested that close cooperation between otorhinolaryngologist and dentist provides benefit to the patients diagnosed as “high-risk case” for a sinus lift.

## Data Availability

The data used to support the findings of this study are available from the corresponding author upon request.

## References

[1] Boyne P. J., James R. A. (1980). Grafting of the maxillary sinus floor with autogenous marrow and bone. *Journal of Oral Surgery (American Dental Association: 1965)*.

[2] Bhattacharyya N. (1999). Bilateral chronic maxillary sinusitis after the sinus-lift procedure. *American Journal of Otolaryngology*.

[3] Suzuki M., Takahashi K., Kato Y., Baba Y. (2018). A maxillary sinusitis after sinus lift surgery. *Journal of Dentistry & Oral Disorders*.

[4] Lee J. W., Yoo J. Y., Paek S. J. (2016). Correlations between anatomic variations of maxillary sinus ostium and postoperative complication after sinus lifting. *Journal of the Korean Association of Oral and Maxillofacial Surgeons*.

[5] Pignataro L., Mantovani M., Torretta S., Felisati G., Sambataro G. (2008). ENT assessment in the integrated management of candidate for (maxillary) sinus lift. *Acta otorhinolaryngologica Italica: Organo ufficiale della Societa italiana di otorinolaringologia e chirurgia cervico-facciale*.

[6] Torretta S., Mantovani M., Testori T., Cappadona M., Pignataro L. (2013). Importance of ENT assessment in stratifying candidates for sinus floor elevation: A prospective clinical study. *Clinical Oral Implants Research*.

[7] Kunihiro T., Araki Y., Oba T. (2014). Minimally invasive endoscopic middle meatal antrostomy for the prevention of maxillary sinusitis in association with dental implantation in the posterior maxilla—a proposal. *Fukuoka Igaku Zasshi = Hukuoka Acta Medica*.

[8] Tiwana P. S., Kushner G. M., Haug R. H. (2006). Maxillary sinus augmentation. *Dental Clinics of North America*.

